# Bio-Inspired Nitrile Hydration by Peptidic Ligands Based on L-Cysteine, L-Methionine or L-Penicillamine and Pyridine-2,6-dicarboxylic Acid

**DOI:** 10.3390/molecules191220751

**Published:** 2014-12-12

**Authors:** Cillian Byrne, Kate M. Houlihan, Prarthana Devi, Paul Jensen, Peter J. Rutledge

**Affiliations:** School of Chemistry F11, The University of Sydney, Sydney, NSW 2006, Australia; E-Mails: cilliandbyrne@googlemail.com (C.B.); tynan.kate@gmail.com (K.M.H.); prarthana.devi@sydney.edu.au (P.D.); paul.jensen.email@gmail.com (P.J.)

**Keywords:** nitrile hydratase, peptidomimetics, metalloenzyme, amino acid

## Abstract

Nitrile hydratase (NHase, EC 4.2.1.84) is a metalloenzyme which catalyses the conversion of nitriles to amides. The high efficiency and broad substrate range of NHase have led to the successful application of this enzyme as a biocatalyst in the industrial syntheses of acrylamide and nicotinamide and in the bioremediation of nitrile waste. Crystal structures of both cobalt(III)- and iron(III)-dependent NHases reveal an unusual metal binding motif made up from six sequential amino acids and comprising two amide nitrogens from the peptide backbone and three cysteine-derived sulfur ligands, each at a different oxidation state (thiolate, sulfenate and sulfinate). Based on the active site geometry revealed by these crystal structures, we have designed a series of small-molecule ligands which integrate essential features of the NHase metal binding motif into a readily accessible peptide environment. We report the synthesis of ligands based on a pyridine-2,6-dicarboxylic acid scaffold and L-cysteine, L-*S*-methylcysteine, L-methionine or L-penicillamine. These ligands have been combined with cobalt(III) and iron(III) and tested as catalysts for biomimetic nitrile hydration. The highest levels of activity are observed with the L-penicillamine ligand which, in combination with cobalt(III), converts acetonitrile to acetamide at 1.25 turnovers and benzonitrile to benzamide at 1.20 turnovers.

## 1. Introduction

**Figure 1:**
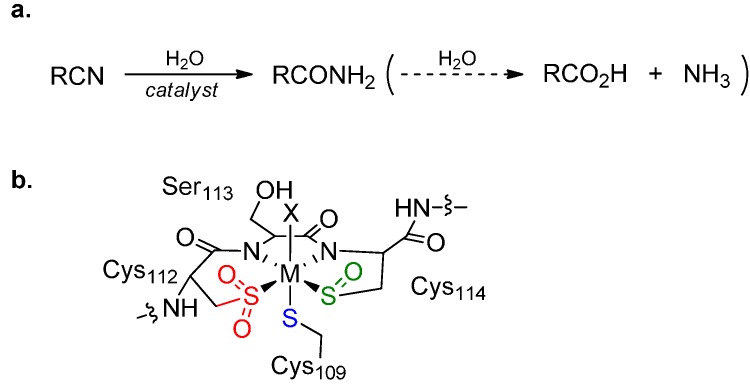
(**a**) Nitrile hydration to the primary amide (and onward to carboxylic acid and ammonia); (**b**) The metal binding environment at the active site of Co-NHase [22]. The metal is coordinated by two nitrogen atoms from backbone amides and three sulfur atoms; all the *S*-ligands derive from the side-chain thiols of cysteine residues, but each is in a different oxidation state: thiolate (blue), sulfenate (green) and sulfinate (red). X = solvent.

The development of mild, environmentally benign methods to convert organic nitriles to primary amides (Figure 1a) is of considerable interest given potential applications of these methods in synthesis, bioremediation and the chemical industry [1,2,3]. Catalytic hydration of nitriles offers an efficient route to amides, which have a wide range of potential applications in polymer manufacture and pharmaceutical synthesis [1,2].

Classical methods for hydrating nitriles require treatment with strong acid or base under forcing conditions which often result in over-hydrolysis to the carboxylic acid [3]. A number of promising systems have been developed in recent years using transition metal catalysis to effect nitrile hydration under increasingly mild and selective conditions [1,2]. Of particular note are recent approaches employing ruthenium [4,5,6,7], rhodium [8,9], palladium [10,11], platinum [12,13], copper [14], silver [15] and gold catalysts [16,17]. Transition metal-free strategies have also been reported recently using potassium carbonate [18] or 1,3-dimethylimidazolium hydrogen carbonate as an organocatalyst [19], and anhydrous conversion of nitriles to amides has been achieved using aldoximes as the water source [11,20,21].

Biocatalysis offers another efficient route to amides from nitriles [23,24]. The hydro-lyase enzyme nitrile hydratase (NHase, EC 4.2.1.84) is used in the industrial production of nicotinamide and acrylamide [24,25], and is increasingly utilised as a biocatalyst for synthesis [26]. NHase has been well studied, and a variety of mechanistic investigations and high-resolution crystal structures reported [22,23,27,28]. NHase is a metalloenzyme and incorporates iron(III) or cobalt(III) in an unusual coordination sphere (Figure 1b). It was long thought that the active site metal ion either activated water to attack the nitrile or activated the nitrile to attack by water [29,30]. However recent structural studies by Holz and co-workers [31] and theoretical calculations by Hopman and Himo [32,33] suggest that the cysteine-sulfenic acid ligand is the catalytic nucleophile.

**Figure 2:**
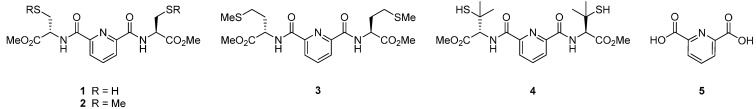
Ligands **1**–**4** prepared and studied in the current investigation are based on pyridine-2,6-dicarboxylic acid **5** and sulfur-containing amino acids.

Several groups have used NHase as the inspiration for biomimetic nitrile hydration [29,30,34,35,36,37,38,39,40]. A handful of synthetic small-molecule complexes incorporating nitrogen and sulfur ligands have been shown to bind nitriles [41] and to promote nitrile hydration in solution [4,35,37,39]. Building on these previous biomimetic approaches to nitrile hydration and our work with related non-heme iron systems [42,43,44,45,46,47], we report now the synthesis and evaluation of ligands **1**–**4** based on the pyridine-2,6-dicarboxylic acid scaffold **5** (Figure 2) and the amino acids l-cysteine (**6**), l-*S*-methylcysteine (**7**), l-methionine (**8**) and l-penicillamine (**9**). These compounds introduce a peptide character around the pyridine-2,6-dicarboxamide core to furnish pentadentate N_3_S_2_ ligands to mimic the NHase active site.

## 2. Results and Discussion

### 2.1. Synthesis of Ligands

**Scheme 1:**
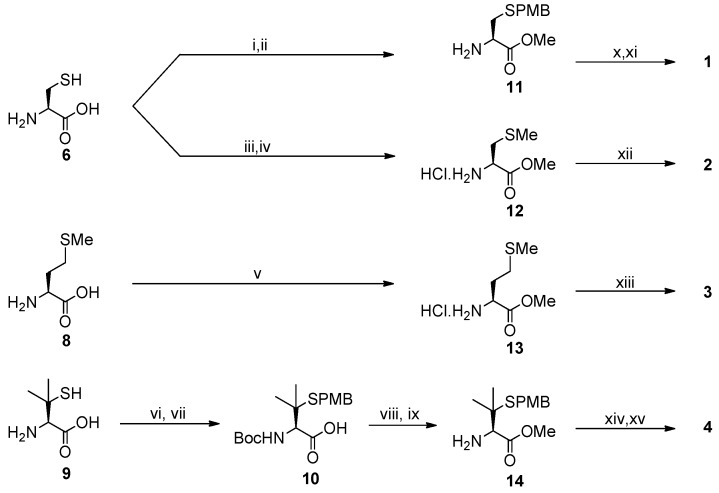
Synthesis of ligands **1**–**4** from pyridine-2,6-dicarboxylic acid (**5**) and the amino acids l-cysteine (**6**), l-*S*-methylcysteine (**7**), l-methionine (**8**) and l-penicillamine (**9**).

Ligands **1**–**4** were prepared from pyridine-2,6-dicarboxylic acid (**5**) and l-cysteine (**6**), l-methionine (**8**) and l-penicillamine (**9**) (Scheme 1). The amino acid constituents were derivatised using established procedures for the preparation of *S*-methyl-l-cysteine [48] and *S*-*p*-methoxybenzyl-l-cysteine [49] from l-cysteine (**6**), protection of l-penicillamine (**9**) via the *S*-*p*-methoxybenzyl (PMB) derivative **10** [49], and methyl esterification [50,51]. The methyl esters **11**–**14** were then coupled to the diacid **5** using *N,N,N′,N′*-tetramethyl-*O*-(1*H*-benzotriazol-1-yl)uronium hexafluorophosphate (HBTU) under standard conditions [52]. This afforded ligands **2** and **3** directly and the *S*-protected forms of ligands **1** and **4**, the *S*-*p*-methoxybenzyl-l-cysteine derivative **15** and *S*-*p*-methoxybenzyl-l-penicillamine analogue **16**. Trifluoroacetic acid-mediated deprotection using triisopropylsilane (TIS) as a cation scavenger was used to convert **15** to ligand **1** and **16** to ligand **4** [49]. The methionine derivative **3** has previously been reported by Deardau *et al*., who investigated the use of this compound and the pyridine *N*-oxide derivative as a ligand for enantioselective reduction of ketones [53], while compound **1** has been described briefly (without full characterisation details) in a report detailing its use in the preparation of hybrid membranes that incorporate amide receptors [54].

*Reagents and Conditions*: i. PMBCl, TFA, DCM, 0 °C 1 h then rt 18 h, 93%; ii. SOCl_2_, MeOH, reflux, 20 h, 77%; iii. Na, MeI, EtOH, 0 °C, 30 min, 85%; iv. SOCl_2_, MeOH, reflux 4 h then rt 16 h, 100%; v. SOCl_2_, MeOH, rt 4 h then reflux 20 h, 89%; vi. PMBCl, AcOH, 80 °C, 72 h; vii. Boc_2_O, Et_2_^i^PrN, MeOH, reflux 3 h then rt 16 h, 89% over two steps; viii. NaHCO_3_, MeI, DMF, rt, 16 h, 62%; ix. *p*-toluenesulfonic acid, CH_2_Cl_2_, reflux, 3 h then NaHCO_3_, 90%; x. pyridine-2,6-dicarboxylic acid **5**, HBTU, Et_3_N, CH_2_Cl_2_, rt, 20 h, 42%; xi. TFA, TIS, reflux, 2.5 h, 63%; xii. **5**, HBTU, Et_3_N, CH_2_Cl_2_, rt, 23 h, 68%; xiii, **5**, HBTU, Et_3_N, CH_2_Cl_2_, 24 h, rt, 33%; xiv, **5**, HBTU, Et_2_^i^PrN, CHCl_3_, rt, 24 h 58%; xv. TFA, TIS, reflux, 3.5 h 91%.

**Figure 3:**
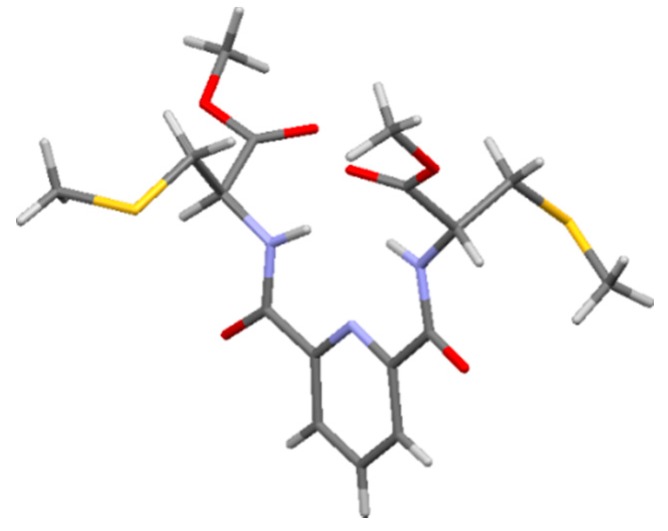
X-Ray crystal structure of pyridine-2,6-dicarboxylic acid bis(*S*-methyl-l-cysteine methyl ester)carboxamide (**2**). Carbon atoms are shown in grey, oxygen in red, nitrogen in lilac, sulfur in yellow and hydrogen in white. CCDC-1017988 contains the full supplementary crystallographic data for this paper. These data can be obtained free of charge from The Cambridge Crystallographic Data Centre via http://www.ccdc.cam.ac.uk/data_request/cif.

Crystallisation of compound **2** from methanol/water afforded one colourless plate (0.32 × 0.27 × 0.08 mm) from which a single crystal X-ray structure was determined. This structure reveals *trans* geometry about both amide bonds, and the *S*-methyl groups oriented away from the pyridine nitrogen (Figure 3 and Supporting Information).

### 2.2. Nitrile Hydration Experiments

The ability of ligands **1**–**4** to promote nitrile hydration was assessed under a range of conditions adapted from those reported previously by Chottard and Mascharak to achieve biomimetic nitrile hydration with small-molecule systems [4,35,37,39]. Thus different permutations of ligand **1**–**4**, metal ion (iron(II), iron(III), cobalt(II) and cobalt(III) salts), base (to effect ligand deprotonation and moderate pH), oxidant (to bring about *S*-oxidation) and nitrile substrate were combined *in situ* (Table 1). Acetonitrile **17** was used throughout screening experiments, present in excess as both substrate and co-solvent. Turnover reaction products were analysed by gas chromatography (GC) in comparison to authentic samples of the starting material **17** and expected product acetamide **18**; turnover was quantified using the single point internal standard method [55,56]. See Supporting Information for details of GC conditions and representative chromatograms (Supplementary Figures S1–S4).

**Table 1:** Summary of variables screened in nitrile turnover experiments.

Ligand	Metal Salt	Temperature (°C)	pH	Oxidant
**1** **2** **3** **4**	Fe(OAc)_2 _	0 20 50	2 5 9	+H_2_O_2_ –H_2_O_2_
	Fe(NO_3_)_3 _			
	Na_3_[Co(NO_2_)_6_]			
	CoCl_2_.6H_2_O			
	[Co(NH_3_)_5_Cl]Cl_2_			

Turnover experiments were typically conducted by dissolving the ligand in acetonitrile, treating with aqueous sodium hydroxide (four equivalents for thiolate ligands **1** and **4** (2 × SH, 2 × NH, anticipating that the acidity of the amide protons would be increased by *N*-coordination to the metal); two equivalents for thioethers **2** and **3**) then adding the metal salt to effect complexation. The resulting solution was adjusted to the required pH using hydrochloric acid or aqueous sodium hydroxide solution, and hydrogen peroxide (3 equivalents) was added to one series of experiments with a view to achieving *S*-oxidation *in situ* [34,57]. The mixture was then adjusted to the required temperature and stirred for the allotted time (12–72 h). All turnover reactions were carried out in triplicate in order to obtain consistent results, thus turnover data discussed below are the average of at least three discrete experiments.

A comprehensive range of control experiments was carried out: (i) no metal salt; (ii) no ligand; (iii) no base; and (iv) no oxidant, to test for direct nitrile hydration by the free ligand (thiols are known to increase the rate of nitrile hydrolysis under certain conditions [58]) the metal (since cobalt(III) can directly hydrolyse nitriles in solution [59]), the added base (to control for direct hydrolysis by hydroxide, and by thiolate or metal-bound hydroxide species) and the oxidant (to account for the possibility of direct reaction between nitrile and peroxide). Figure S2 (Supporting Information) shows the chromatogram arising from a typical control experiment. Turnover was only observed when both metal and ligand were present (Table 2).

Ligands **1** and **2** returned no nitrile hydration under most of the conditions screened, and only very low levels of reaction at their best: 0.20 and 0.40 turnovers respectively (*i.e*., sub-stoichiometric amide formation) achieved using Na_3_[Co(NO_2_)_6_]/pH 9/*no* H_2_O_2_/50 °C/72 h with **1** and Fe(NO_3_)_3_/pH 9/H_2_O_2_ (3 eq.)/20 °C/72 h with **2**. The control experiments outlined above confirmed that the specific combinations of ligand, metal, base and oxidant (in the case of **2**) were required to mediate turnover. The methionine ligand **3** did not turn over acetonitrile under any of the conditions tested.

**Table 2:** Summary of optimal conditions for acetonitrile turnover with ligands **1**–**4**.

Ligand	Optimal Turnover Conditions					Turnovers
**1**	Na_3_[Co(NO_2_)]_6_	50 °C	pH 9	72 h	−H_2_O_2_	0.20
**2**	Fe(NO_3_)_3_	20 °C	pH 9	72 h	+H_2_O_2_	0.40
**3**	–	–	–	–	–	–
**4**	Na_3_[Co(NO_2_)]_6_	25 °C	pH 9	12 h	−H_2_O_2_	1.25

**Scheme 2:**
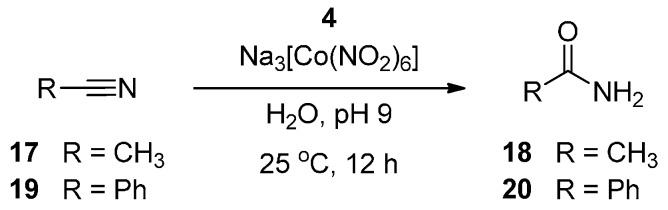
Optimised conditions for nitrile hydration using ligand **4**.

Ligand **4** returned the best turnover results found with these systems, when combined with Na_3_[Co(NO_2_)_6_] and stirred for 12 h at 25 °C and pH 9 (Scheme 2 shows the reaction outcome, Figure S3 (Supporting Information) shows a representative chromatogram for this level of turnover). The geminal dimethyl unit adjacent to the thiolate enhances the sub-stoichiometric turnover seen with **1** to a gently catalytic level of 1.25 turnovers. The addition of peroxide diminishes catalytic activity with this ligand (down to 0.7 turnovers), while increasing reaction temperature to 50 °C also lowers activity to 0.7 turnovers.

To investigate the scope of this ligand, a further set of experiments was carried out using a second substrate, the aromatic nitrile benzonitrile **19**. Under these conditions the combination of ligand **4** and Na_3_[Co(NO_2_)_6_ also turned over benzonitrile **19** as a substrate, with a similar level of conversion to benzamide **20** (1.2 turnovers). (To aid the miscibility of the benzonitrile in water, a small amount of DMF was added to these reactions: 200 µL in a total reaction volume of 7.2 mL).

## 3. Experimental Section

### 3.1. General Information

Chemicals were supplied by Sigma-Aldrich (Castle Hill, NSW, Australia), Merck Millipore (Bayswater, Vic, Australia) or Bio-Scientific (Kirrawee, NSW, Australia). All reactions were carried out under a dry nitrogen or argon atmosphere unless otherwise indicated. Analytical thin-layer chromatography (TLC) was performed on Merck Kieselgel 0.25 mm silica gel 60 F254 plates (Merck Millpore). TLC plates were visualised using UV fluorescence at 254 nm, or by staining with Goofy’s reagent (6:6:194:1 phosphomolybdic acid–conc. sulphuric acid–water–cerium sulfate). Flash chromatography on silica gel was performed using a force flow of the indicated solvent system on Ajax Finechem silica gel (230–400 mesh).

Solvents used for extraction and chromatography were distilled prior to use. Where necessary, solvents and reagents were dried over 4 Å molecular sieves prior to use. Tetrahydrofuran was distilled anaerobically from sodium wire. Dichloromethane, methanol, acetonitrile, diisopropylethylamine and triethylamine were distilled from calcium hydride. Dimethylformamide was distilled and stored over 4 Å molecular sieves. Methyl iodide was purified by passage through neutral alumina. Anisole and benzonitrile and were purified by distillation under reduced pressure. Benzamide was recrystallised from water. All other commercial reagents were used as received without further purification.

Melting points were recorded on a Gallenkamp melting point apparatus (Sanyo Gallenkamp, Loughborough, UK). ^1^H- and ^13^C-NMR spectra were recorded on Bruker Avance DPX200, DPX300 and DPX400 spectrometers (Bruker, Billerica, MA, USA). Chemical shifts (δ) are reported in ppm from tetramethylsilane (TMS), using TMS or the solvent resonance as the internal reference. Low resolution mass spectra were obtained on a Finnigan LCQ MS Detector (ESI, APCI) (Thermo Fisher Scientific, Waltham, MA, USA). High resolution mass spectra were obtained on a Fisons VG Tofspec (MALDI-TOF) (Thermo Fisher Scientific). Optical rotations were measured on a Bio-Rad FTS-40 polarimeter (Bio-Rad Laboratories Inc., Hercules, CA, USA) with sodium lamp operating at 589 nm using a 1 dm cell and concentrations (c) measured in 100 mg/mL. IR spectra were measured at room temperature on a Bio-Rad Shimadzu 8400S (Bio-Rad Laboratories Inc.) using a sodium chloride cell, NaCl plates or KBr disc. Wavenumbers are given in reciprocal cm and bands are expressed as strong (s), medium (m) and weak (w). Gas chromatography was carried out on a Hewlett Packard 5890A gas chromatograph (Hewlett Packard, Palo Alto, CA, USA). The column used was a SGE 25QC2/BPX with an internal diameter of 0.25 nm.

### 3.2. Preparation of Protected Amino Acids

#### *General Methyl Esterification Procedure* [50]

Amino acids/protected amino acids were treated with thionyl chloride (2 equivalents) in methanol, heated at reflux for 4 h and then stirred for 16 h at room temperature. The solvent was removed *in vacuo* and the crude product recrystallized from diethyl ether to yield the methyl ester as its hydrochloride salt in high yield and high purity.

*S-p-Methoxybenzyl-l-cysteine methyl ester* (**11**) [49,50]. l-Cysteine (**6**, 5.61 g, 46.3 mmol) was dissolved in TFA (15 mL) and DCM (50 mL), cooled to 0 °C and stirred while a solution of *p-*methoxybenzyl chloride (7.22 g, 46.1 mmol) in DCM (50 mL) was added dropwise. The reaction mixture was stirred at 0 °C for 1 h then allowed to warm to room temperature and stirred for 18 h. Methanol (20 mL) was added and the product was extracted into water (2 × 70 mL). The aqueous phase was washed with DCM (70 mL) and solid impurities removed by filtration. The filtrate was concentrated *in vacuo* to afford *S*-*p-*methoxybenzyl-l-cysteine trifluoroacetate salt as a white solid (15.24 g, 93%); this intermediate (15.24 g, 42.9 mmol) was dissolved in methanol (100 mL) and thionyl chloride (5.0 mL, 68.5 mmol) added drop-wise. The reaction mixture was heated at reflux for 18 h before the solvent was removed *in vacuo* to yield an off-white solid. This crude product was partitioned between ethyl acetate (40 mL) and saturated aqueous NaHCO_3_ (20 mL). The organic phase was washed with a further aliquot of saturated aqueous NaHCO_3_ (20 mL), water (40 mL) and brine (40 mL) then dried (MgSO_4_). The solvent was removed *in vacuo* to yield **11** as viscous brown oil (8.42 g, 77%); ν_max_ (thin film): 3364, 2950, 2837, 2363, 1734, 1643, 1551, 1450, 1300; δ_H_ (CDCl_3_, 300 MHz) 2.70 (1H, dd, *J* = 13.5, 7.5), 2.84 (1H, dd, *J* = 13.5, 4.5), 3.70 (2H, s), 3.72 (3H, s), 3.79 (3H, s), 4.62–4.68 (1H, m), 5.31 (1H, br s), 6.84 (2H, d, *J* = 8.5), 7.22 (2H d, *J* = 8.5); δ_C_ (CDCl_3_, 75 MHz) 36.0, 36.3, 52.7, 54.0, 55.3, 114.0, 130.0, 150.2, 158.7, 174.4; *m/z* (ESI+) 256 (100%, [M+H]^+^). Data in agreement with literature [60].

*S-Methyl-l-cysteine methyl ester hydrochloride salt* (**12**) [48]. l-Cysteine (**6**, 4.20 g, 35.0 mmol) was dissolved in absolute ethanol (90 mL). Sodium metal (2.75 g, 120.0 mmol) was added slowly to the solution at 0 °C. The reaction mixture was allowed to stir for a further 30 min. Methyl iodide (2.10 mL, 26.0 mmol, purified by passing down a short column of alumina prior to use) was added drop-wise to the reaction at 0 °C. The mixture was allowed to warm to room temperature over a period of 30 min. The solution was adjusted to pH 5 using HCl (10 m) before diethyl ether (80 mL) was added, affording a cloudy white suspension. The mixture was filtered under vacuum and the isolated solid was washed with ether (100 mL) and dried under vacuum to give *S*-methyl-l-cysteine (4.00 g, 85%). A portion of this solid (0.56 g, 4.40 mmol) was subjected to the general methyl esterification conditions using thionyl chloride (0.65 mL, 8.80 mmol) in methanol (10 mL) to give the title compound **12** as a white solid (0.82 g, 85%); ν_max_ (KBr pellet): 3362 (s), 1745 (s), 1606 (s); δ_H_ (300 MHz, D_2_O): 2.10 (3H, s), 3.01 (1H, dd, *J =* 15.0, 7.5 Hz), 3.13 (1H, dd, *J* = 15.0, 4.5 Hz), 3.81 (3H, s), 4.30 (1H, dd, *J =* 7.5, 4.5 Hz); δ_C_ (75.4 MHz, CDCl_3_): 16.2, 34.1, 52.7, 53.6, 168.8; *m/z* (ES^+^): 150 (15%, [M+H]^+^). Data in agreement with literature [61].

*l-Methionine methyl ester hydrochloride salt* (**13**) [50]. Using the general methyl esterification conditions, l-methionine (**8**, 2.00 g, 13.4 mmol) was treated with thionyl chloride (2.00 mL, 26.8 mmol) in methanol (20 mL) to afford the title compound as a white powder (1.95 g, 89%); ν_max_ (KBr pellet): 3080 (s), 1747 (s), 2840 (w), 1236 (s); δ_H_ (300 MHz, CDCl_3_): 2.28 (3H, s), 2.28–2.33 (2H, m), 2.67–2.77 (2H, m), 3.87 (3H, s), 4.16–4.30 (1H, m); δ_C_ (75.4 MHz, CD_3_OD): 15.4, 30.5, 31.1, 53.2, 54.2, 171.2; *m/z* (ES^+^): 164 (100%, [M+H]^+^). Data in agreement with literature [62].

*S-p-Methoxybenzyl-l-penicillamine methyl ester* (**14**) [51]. To a solution of l-penicillamine (**9**, 0.25 g, 1.60 mmol) in glacial acetic acid (5.0 mL) was added *p*-methoxybenzyl chloride (0.24 mg, 227 μL, 1.55 mmol). The resulting mixture was heated at 80 °C for 72 h then the solvent was removed *in vacuo*. The oily residue was dissolved in acetonitrile (5.0 mL) and the solvent was removed *in vacuo*. This procedure was repeated twice more to reveal a white solid. This solid was dissolved in a solution of diisopropylethylamine in methanol (10% v/v, 12.0 mL) and stirred. Di-*tert-*butyl-dicarbonate (0.71 g, 3.25 mmol) was added in a single portion. The reaction mixture was heated at reflux for 3 h and then left to stir at room temperature overnight. The solvent was removed *in vacuo* and the oily residue was dissolved in ethyl acetate (50 mL). This solution was washed with aqueous HCl (1 m, 40 mL), water (40 mL) and brine (40 mL), then dried over MgSO_4_, and the solvent was removed *in vacuo* to reveal *N*-*tert*-butyloxycarbonyl-*S*-*p*-methoxybenzyl-l-penicillamine (**10**) as a white solid (0.53 g, 89%); δ_H_ (300 MHz, CD_3_CN): 1.34 (3H, s), 1.41 (3H, s), 1.42 (9H, s), 3.77 (3H, s), 3.73–3.75 (2H, m), 4.20 (1H, br d, *J =* 8.5 Hz), 5.62 (1H, br d , *J =* 8.5 Hz), 6.85 (2H, d, *J* = 8.5 Hz), 7.23 (2H, d, *J =* 8.5 Hz); δ_C_ (75.4 MHz, CD_3_CN): 25.6, 27.1, 28.6, 33.1, 48.1, 56.2, 61.2, 80.2, 114.7, 130.6, 131.2, 159.8, 168.2, 172.2; *m*/*z* (ES^−^): 368 ([M–H]^−^, 90%), 737 ([2M–H]^−^, 100%). Compound** 10 **(1.55 g, 4.21 mmol) and NaHCO_3_ (0.71 g, 8.40 mmol) were dissolved in DMF (20 mL) and stirred while methyl iodide (1.20 g, 524 μL, 8.40 mmol) was added. The mixture was stirred overnight. The solvent was removed *in vacuo* and the crude residue redissolved in ethyl acetate (20 mL) and washed with saturated aqueous NaHCO_3_ (10 mL), water (10 mL) and brine (10 mL). The organic phase was dried over MgSO_4_ and the solvent removed *in vacuo*. The residue was combined with *p*-toluenesulfonic acid (0.54 g, 4.20 mmol) in dichloromethane (25 mL) and heated at reflux for 4 h. The resulting solution was cooled to room temperature then washed with saturated aqueous NaHCO_3_ (10 mL), water (10 mL) and brine (10 mL). The organic phase was dried over MgSO_4_ and evaporated *in vacuo* to yield the title compound as a clear oil (0.70 g, 59%); ${\left[\alpha \right]}_{D}^{20}$ = +90.4 (*c* = 0.66 in EtOAc); ν_max_ (thin film) 3378 (s), 1740 (s), 834 (s); δ_H_ (300 MHz, CDCl_3_): 1.29 (3H, s), 1.42 (3H, s), 1.77 (2H, s), 3.49 (1H, s), 3.71 (1H, s), 3.73 (3H, s), 3.76 (1H, s), 3.78 (3H, s), 6.83 (2H, d, *J =* 5.5 Hz), 7.23 (2H, d, *J =* 5.5 Hz); δ_C_ (75.4 MHz, CDCl_3_): 24.0 26.6, 32.8, 50.2, 51.9, 55.4, 61.8, 110.8, 114.2, 130.2, 159.0, 173.9; *m/z* (ES+): 284 ([M+H]^+^, 100%); HRMS (ES^+^): [M+H]^+^ C_14_H_22_NO_3_S requires 284.1315, found 284.1325.

### 3.3. Peptide Coupling Method

Amino acid methyl ester (as free amine or HCl salt, 0.2–1.8 g, 2 eq.) pyridine-2,6-dicarboxylic acid (**5**, 1 eq.) and *N,N,N*′*,N*′*-*tetramethyl-*O-(*1*H*-benzotriazol-1-yl)uronium hexafluorophosphate (HBTU, 2 eq.) were dissolved in DCM or chloroform (20–40 mL) and triethylamine or DIPEA (2 eq. for free amine, 4 eq. for HCl salt) was added. The reaction mixture was stirred at room temperature for 22–48 h while monitored by TLC. Additional DCM or chloroform (10–20 mL) was added and the solution washed with equivalent volumes of water, 1 M hydrochloric acid, saturated aqueous sodium hydrogen carbonate, and brine. The organic phase was dried over MgSO_4_ then concentrated *in vacuo* to give the crude product (generally a yellow oil) which was purified by column chromatography.

### 3.4. Ligand Synthesis

*Pyridine-2,6-dicarboxylic acid bis(S-p-methoxybenzyl-l-cysteine methyl ester) carboxamide* (**15**). Pyridine-2,6-dicarboxylic acid (**5**, 0.06 g, 0.39 mmol) and *S*-*p*-methoxybenzyl-l-cysteine methyl ester (**11**, 0.20 g, 0.78 mmol) were coupled to give **15** as a thick, colourless oil (0.21 g, 42%) after purification by column chromatography (EtOAc‒petroleum ether 2:1); R_f_ 0.30 (EtOAc‒petroleum ether 2:1); ${\left[\alpha \right]}_{D}^{20}$ = −44.8 (*c =* 0.5, CHCl_3_); ν_max_ (CHCl_3_): 3501 (w), 1742 (s), 1736 (s); δ_H_ (300 MHz, CDCl_3_): 3.01 (4H, d, *J* = 5.5 Hz), 3.62–3.84 (16H, overlapping m and 2 × s), 4.96 (2H, dd, *J* = 18.5, 7.5 Hz), 6.77 (4H, d, *J* = 8.5 Hz), 7.20 (4H, d, *J* = 8.5 Hz), 8.05 (1H, t, *J* = 7.5 Hz), 8.35 (2H, d, *J* = 7.5 Hz), 8.57 (2H, d, *J* = 7.5 Hz); δ_C_ (75.4 MHz, CDCl_3_): 33.2, 36.0, 51.7, 52.7, 55.2, 113.9, 125.3, 129.4, 130.0, 139.1, 148.2, 158.7, 163.1, 171.0; *m/z* HRMS (ES^+^): C_31_H_35_N_3_O_8_S_2_Na^+^ ([M+Na]^+^) requires 664.1763, found 664.1761.

*Pyridine-2,6-dicarboxylic acid bis(S-methyl-l-cysteine methyl ester)carboxamide* (**2**). Pyridine-2,6-dicarboxylic acid (**5**, 0.23 g, 1.4 mmol) and *S*-methyl-l-cysteine methyl ester hydrochloride salt (**12**, 0.50 g, 2.7 mmol) were combined to give **2** as a pale yellow oil (0.39 g, 68%) after purification by column chromatography (EtOAc‒cyclohexane 2:1); R_f_ 0.40 (EtOAc‒cyclohexane 2:1); ${\left[\alpha \right]}_{D}^{20}$ = −8.0 (*c =* 1.0, CHCl_3_); ν_max_ (thin film) 3354 (s), 3043 (w), 1743 (s), 1678 (s); δ_H_ (300 MHz, CDCl_3_): 2.19 (6H, s), 2.84 (4H, d, *J =* 3.5 Hz), 3.64 (6H, s), 5.02 (2H, dt, *J* = 8.0, 5.5 Hz), 8.07 (1H, t, *J* = 8.0 Hz), 8.37 (2H, d, *J* = 8.0 Hz), 8.64 (2H, d *J* = 8.0 Hz); δ_C_ (75.4 MHz, CDCl_3_): 16.1, 36.4, 51.6, 52.8, 125.8, 139.5, 148.7, 163.6, 171.4; *m/z* (ES^+^): 452 (30%, [M+Na]^+^), 430 (100%, [M+H]^+^); HRMS (ES^+^): C_17_H_24_N_3_O_6_S_2_ ([M+H]^+^) requires 430.1107, found 430.1118.

*Pyridine-2,6-dicarboxylic acid bis(l-methionine methyl ester)carboxamide* (**3**). Pyridine-2,6-dicarboxylic acid (**5**, 0.92 g, 5.5 mmol) and l-methionine methyl ester hydrochloride salt (**13**, 1.80 g, 11.0 mmol) were united to give **3** as a yellow oil (0.81 g, 33%) after purification by column chromatography (DCM: EtOAc 4:1); R_f_ 0.30 (DCM: EtOAc 4:1); ${\left[\alpha \right]}_{D}^{20}$ = −10.3 (*c =* 1.0, CHCl_3_); ν_max_ (thin film): 3392 (s), 1739 (s), 1677 (s); δ_H_ (300 MHz, CDCl_3_): 2.08 (6H, s), 2.12–2.24 (4H, m), 3.05–3.16 (4H, m), 3.74 (6H, s), 4.87–4.89 (2H, m), 7.92–8.02 (1H, t, *J* = 7.5 Hz), 8.29 (2H, d, *J* = 7.5 Hz), 8.57 (2H, d, *J* = 8.0 Hz); δ_C_ (75.4 MHz, CDCl_3_): 15.7, 30.6, 31.5, 52.3, 52.9, 125.9, 139.1, 147.0, 163.7, 172.9; *m/z* (ES^+^): 458 (100%, [M+H]^+^); HRMS (ES^+^): C_19_H_28_N_3_O_6_S_2_ ([M+H]^+^) requires 458.1417, found 458.1409. Data in agreement with literature [54].

*Pyridine-2,6-dicarboxylic acid bis(S-p-methoxybenzyl-l-penicillamine methyl ester)* *carboxamide* (**16**). Pyridine-2,6-dicarboxylic acid (**5**, 0.16 g, 0.97 mmol) and *S*-*p*-methoxybenzyl-l-penicillamine methyl ester (**14**, 0.55 g, 1.94 mmol) were joined to give **16** as a clear yellow oil (0.39 g, 58%) after purification by column chromatography (hexane‒EtOAc 6:4); R_f_ 0.25 (hexane‒EtOAc 6:4); ${\left[\alpha \right]}_{D}^{20}$ = +2.1 (*c =* 1.12, EtOAc); ν_max_ (KBr): 3401 (m), 1742 (m), 1681 (m); δH (300 MHz, CDCl3): 1.26 (6H, s), 1.48 (6H, s), 3.62 (6H, s), 3.73 (2H, s), 3.74 (1H, s), 3.76 (6H, s), 4.75 (2H, d, *J =* 7.5 Hz), 6.71 (4H, d, *J =* 8.5 Hz), 7.18 (4H, d, *J =* 8.5 Hz), 8.05 (1H, t, *J =* 7.5 Hz), 8.35 (2H, d, *J =* 8.0 Hz), 8.62 (2H, d, *J =* 7.5 Hz); δC (75.4 MHz, CDCl3): 25.0, 27.7, 33.4, 48.8, 52.8, 55.6, 60.5, 114.6, 126.3, 129.3, 131.1, 139.2, 148.3, 160.0, 164.8, 171.5; *m/z* (ES+): 720 ([M+Na]+, 100%); HRMS (ES+): C_35_H_44_N_3_O_8_S_2_ ([M+H]^+^) requires 698.2587, found 698.2595.

*Pyridine-2,6-dicarboxylic acid bis(l-cysteine methyl ester)carboxamide* (**1**). Pyridine-2,6-dicarboxylic acid bis(*S*-*p*-methoxybenzyl-l-cysteine methyl ester)carboxamide (**15**, 100 mg, 0.15 mmol) and triisopropylsilane (TIS, 195 μL, 0.95 mmol) were dissolved in TFA (1.25 mL). The reaction mixture was heated at reflux for 2.5 h after which time the solvent was removed *in vacuo*. The residue was washed with ether (3 × 2 mL). The crude product was purified by flash column chromatography (EtOAc‒petroleum ether 2:1) to afford the pure product **1** as white thick oil (41 mg, 63%) which was stored and handled under nitrogen to minimize disulfide formation; R_f _0.20 (ether: hexane 1:1); ${\left[\alpha \right]}_{D}^{20}$ = +8.0 (*c =* 0.1, CHCl_3_); ν_max_ (thin film): 3394 (s), 2570 (w), 1739 (s), 1670 (s); δ_H_ (300 MHz, CDCl_3_): 1.55 (2H, t, *J* = 9.0 Hz), 3.14–3.20 (4H, m), 3.84 (6H, s), 5.07–5.09 (2H, m), 8.08 (1H, t, *J* = 7.5 Hz), 8.37 (2H, d, *J* = 7.5 Hz), 8.66 (2H, d, *J* = 7.0 Hz); δ_C_ (75.4 MHz, CDCl_3_): 27.0, 52.9, 53.6, 125.5, 139.3, 148.2, 163.1, 170.1; *m/z* (ES^+^): 402 (100%, [M+H]^+^), 424 (25%, [M+Na]^+^); HRMS (ES^+^): C_15_H_20_N_3_O_6_S_2_ ([M+H]^+^) requires 402.0791, found 402.0795.

*Pyridine-2,6-dicarboxylic acid bis-(l-penicillamine methyl ester) carboxamide* (**4**). Pyridine-2,6-dicarboxylic acid bis-*S*-*p*-methoxybenzyl-l-penicillamine methyl ester (**16**, 0.40 g, 0.57 mmol) and TIS (720 µL, 3.5 mmol) were dissolved in TFA (5.0 mL). The reaction mixture was heated at reflux for 2.5 h. The solvent was removed *in vacuo* and the residue triturated with ether and hexane. Flash column chromatography of the crude material (hexane‒EtOAc 3:2) furnished **4** as a clear yellow oil (0.24 g, 91%); R_f_ 0.30 (hexane‒EtOAc 3:2); ${\left[\alpha \right]}_{D}^{20}$ = +2.1 (*c =* 0.04, EtOAc); ν_max_ (CHCl_3_): 1743 (s), 1667 (s); δH (300 MHz, CDCl3): 1.52 (6H, s), 1.62 (6H, s), 2.22 (2H, br s), 3.82 (6H, s), 4.83 (2H, d, *J =* 9.5 Hz), 8.06 (1H, t, *J =* 8.0 Hz), 8.34 (2H, d, *J =* 8.0 Hz), 8.75 (2H, d, *J =* 9.5 Hz); δC (75 MHz, CDCl_3_): 29.8, 30.8, 46.7, 52.3, 61.0, 125.6, 139.31, 148.2, 163.2, 170.4; *m/z* (ES+): 480 (10%, [M+Na]^+^); HRMS (ES^+^): C_19_H_27_N_3_NaO_6_S_2_ ([M+Na]^+^) requires 480.1237, found 480.1238.

### 3.5. Crystallography

A colourless plate-like crystal of **2** was attached with Exxon Paratone N, to a short length of fibre supported on a thin piece of copper wire inserted in a copper mounting pin. The crystal was quenched in a cold nitrogen gas stream from an Oxford Cryosystems Cryostream (Oxford Cryosystems Ltd, Long Hanborough, UK). A Bruker-Nonius FR591 Kappa APEX II diffractometer (Bruker Analytical X-ray Instruments Inc., Madison, WI, USA) employing graphite monochromated MoKα radiation generated from a fine-focus rotating anode was used for the data collection. Cell constants were obtained from a least squares refinement against 7789 reflections located between 5.68 and 65.16° 2θ. Data were collected at 150(2) Kelvin with ϕ and ω scans to 65.18° 2θ. The data integration and reduction were undertaken with SAINT (Bruker Analytical X-ray Instruments Inc.) and XPREP (Bruker Analytical X-ray Instruments Inc.) [63], and subsequent computations were carried out with the X-Seed graphical user interface (University of Missouri, Columbia, MO, USA) [64]. An empirical absorption correction determined with SADABS (University of Göttingen, Göttingen, Germany) was applied to the data [65,66].

The structure was solved in the space group P1(#1) by direct methods with SHELXS-97 (University of Göttingen), and extended and refined with SHELXL-97 [67]. The non-hydrogen atoms in the asymmetric unit were modelled with anisotropic displacement parameters. A riding atom model with group displacement parameters was used for the hydrogen atoms. The absolute structure was established with the Flack parameter [68,69,70,71] refining to −0.01(3).

### 3.6. Turnover Experiments

#### 3.6.1. General Procedure

The ligand (0.10 mmol) was dissolved in the nitrile (5.0 mL) and sodium hydroxide (2–4 eq., 0.20–0.40 mmol) was added. The metal salt (0.10 mmol) was added and the mixture stirred at ambient temperature for 15 min. If oxidant was to be included, the solution was cooled to 0 °C while H_2_O_2_ (30%, 0.30 mmol) was added dropwise with stirring, and the mixture was stirred on ice for 30 min. Water (2.0 mL) was added and the mixture adjusted to the required pH (2, 5 or 9, using aqueous HCl or NaOH) and temperature (0, 20 or 50 °C) then stirred for 12–72 h. The solvent was removed *in vacuo*, the residue re-dissolved in acetonitrile (1 mL) and the solution filtered through a plug of silica to remove particulates. The sample was analysed by gas chromatography against an internal standard, and product(s) were identified by comparison to authentic samples.

#### 3.6.2. GC Analysis

Calibration of the internal standard/ relative response factor was carried out using the single point internal standard method [55,56]. Thus equimolar quantities of anisole and acetamide in ethyl acetate (1 μL) were injected into the GC and the peak area for each was recorded. Three injections were made and an average of the three measurements taken. Under the conditions used (Table S1, Supporting Information) residual acetonitrile **17** eluted with the solvent front. Anisole, the internal standard, eluted with a retention time (R_t_) of 6.88 ± 0.03 min, while the product acetamide **18** eluted at R_t_ 12.64 ± 0.06 min (Figure S1). The relative response factor (the relative peak area of the two components) was 3.35 (*i.e*., the area of the internal standard was 3.35 times greater than the area of the acetamide product when present in equal molar quantities). By adding a defined quantity of anisole to turnover reaction mixtures immediately prior to analysis by GC and using the relative response factor (3.35), the amount of acetamide formed could be accurately quantified, even at <0.1 turnovers. Further details of GC run times and temperatures, calculation of internal response factors and example chromatograms are provided in the Supporting Information (Tables S1 and S2, Figures S1–S4).

## 4. Conclusions

We have prepared a series of pentadentate ligands that combine sulfur-containing amino acids on a pyridine-2,6-dicarboxylic acid scaffold to render an N_3_S_2_ donor set incorporating key elements of the NHase active site. Evaluating the iron(III)- and cobalt(III)-complexes of these ligands as catalysts for nitrile hydration demonstrates low levels of turnover (1.20–1.25 turnovers) for the penicillamine ligand **4** in combination with cobalt(III), with both acetonitrile and benzonitrile substrates. Placed in context, the most active small-molecule mimics of NHase catalysis to date employ preformed cobalt complexes under optimised conditions to achieve 15–18 turnovers for a non-oxidised pyridine dithiolate ligand (at 50 °C and pH 9.5 in Tris buffer) [35], and 50 turnovers with a bis-sulfenate ligand (at 4 °C and pH 4.8 in acetate buffer) [37]. Future work will investigate the importance of the pyridine nitrogen to the catalytic activity of ligand **4**.

## Supplementary Materials

Supplementary materials can be accessed at: http://www.mdpi.com/1420-3049/19/12/20751/s1.
