# Supplementary material for: Bio-Inspired Nitrile Hydration by Peptidic Ligands Based on L-Cysteine, L-Methionine or L-Penicillamine and Pyridine-2,6-dicarboxylic Acid
Source: Molecules. 2014 Dec 12;19(12):20751–67. doi: 10.3390/molecules191220751 (PMC6271992; doi:10.3390/molecules191220751)
Supplement: Supplementary file 1 [file molecules-19-20751-s001.pdf]

Supplementary Materials

1. Nitrile Turnover Experiments

1.1. Quantification of Acetonitrile Turnover

The extent of nitrile hydration/amide generation was determined using gas chromatography (GC). GC conditions were developed to achieve baseline separation of the amide from the internal standard and any by-products (Table S1). Under these conditions, acetonitrile **17** elutes with the solvent front, the internal standard anisole at $R_t = 6.88 \pm 0.03$ min and acetamide **18** at $R_t = 12.64 \pm 0.06$ min (Figure S1).

Table S1. Gradient GC elution conditions for acetamide determination.

Time (min)	Temp/Rate Change	Time	Final Temperature
0–1	60 °C	1 min	60 °C
1–11	10 °C/min	10 min	160 °C
11–13	20 °C/min	2 min	200 °C
13–18	No change	5 min	200 °C

The amount of acetamide formed in each turnover reaction was quantified using the single point internal standard method [1,2]. High purity standards were first used to quantify the relative response factors of the internal standard and the amide product (Figure S1), using this equation to calculate the internal response factor (IRF):

$$\text{IRF} = \frac{\text{area}_{\text{internal standard}} \times \text{amount}_{\text{specific compound}}}{\text{amount}_{\text{internal standard}} \times \text{area}_{\text{specific compound}}}$$

By adding a defined quantity of anisole to the turnover reaction mixture immediately prior to GC analysis, the amount of acetamide formed in each turnover experiment can then be calculated as:

$$\text{amount}_{\text{acetamide}} = \frac{\text{amount}_{\text{anisole}} \times \text{area}_{\text{acetamide}} \times \text{IRF}}{\text{area}_{\text{anisole}}}$$

1.2. Example Chromatograms Showing Acetonitrile Turnover

1.2.1. Calibration of Authentic Product Sample

Figure S1. Gas chromatogram of calibration sample containing high-purity acetamide **18** ($R_t = 12.64 \pm 0.06$ min) and the internal standard anisole ($R_t = 6.88 \pm 0.03$). Acetonitrile **17** elutes with the solvent front.

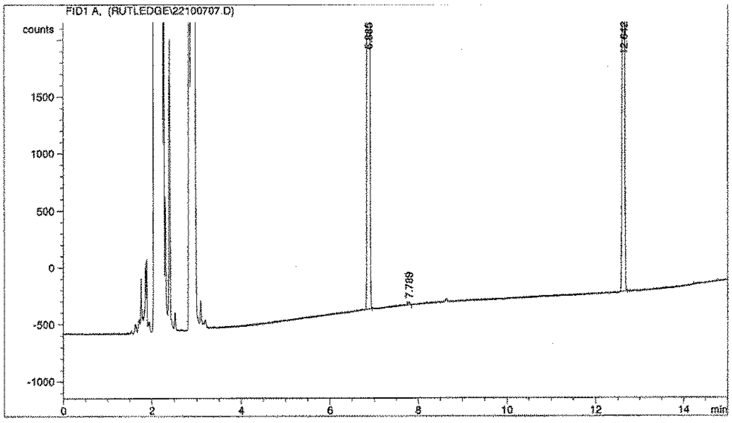

1.2.2. Representative Control Experiment

Figure S2. Gas chromatogram of representative control experiment: 'no ligand' control (reaction of acetonitrile **17** with $Na_3[Co(NO_2)_6]$ only). Note the absence of acetamide **18** at R_t 12.64 ± 0.06.

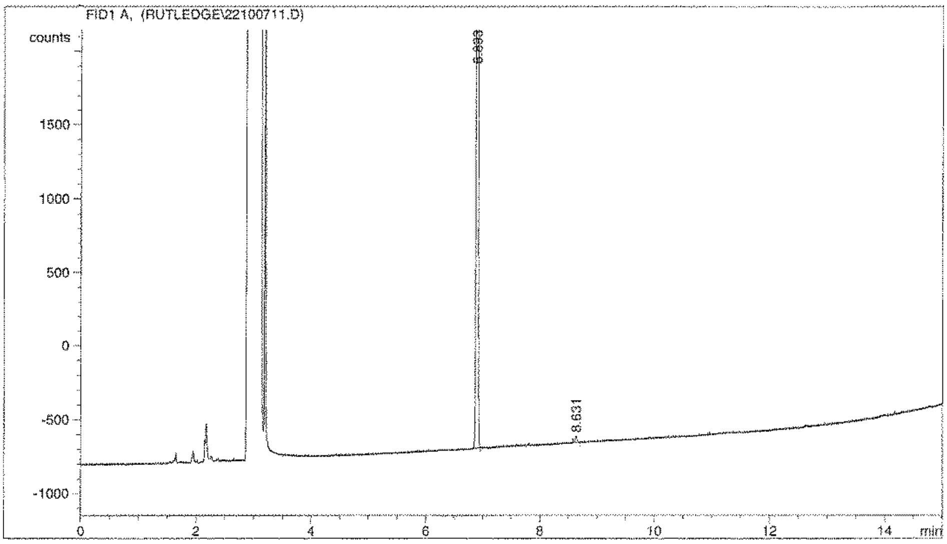

1.2.3. Representative "Low Turnover" Reaction

Figure S3. Gas chromatogram of representative 'low turnover' reaction (reaction of acetonitrile **17** with the combination of ligand **4**, $Na_3[Co(NO_2)_6]$ and H_2O_2 at 50 °C): the trace acetamide peak at R_t 12.58 corresponds to <0.1 turnovers, but can still be discerned and quantified relative to the anisole signal at 6.86 min using the single point internal standard method.

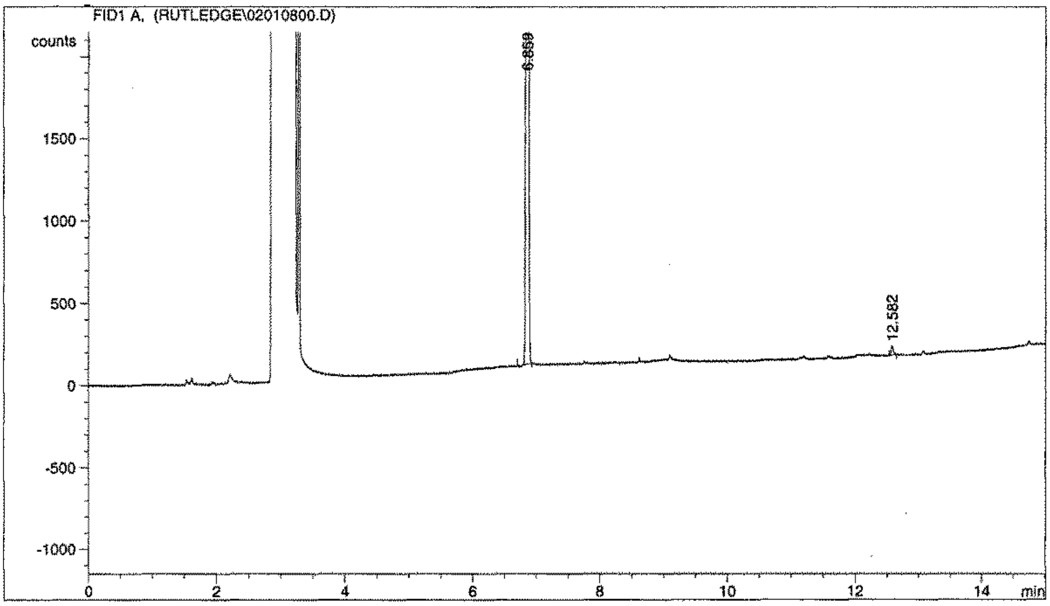

1.2.4. Representative "High Turnover" Reaction

Figure S4. Gas chromatogram of representative "high turnover" reaction (reaction of acetonitrile **17** with the combination of ligand **4** and $Na_3[Co(NO_2)_6]$ at pH 9 and room temperature—see Table 2 and Scheme 2): the acetamide peak at R_t 12.58 is again quantified relative to the anisole signal at 6.88 min using the single point internal standard method.

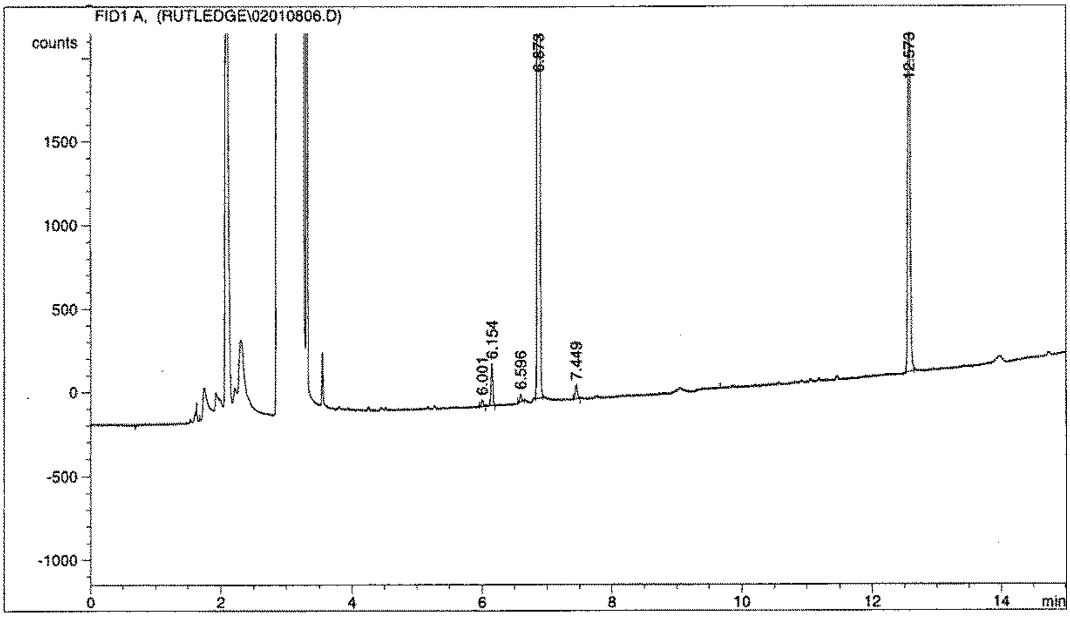

1.3. GC Conditions for Assaying Benzonitrile Turnover

Quantification of benzamide **20** formation required slightly higher GC temperatures than for acetamide **18**. Under these conditions, benzamide **20** eluted with a retention time of 22.83 ± 0.03 min and the anisole internal standard with a retention time of 4.95 ± 0.01 min. The relative response factor was 1.14. Analysis of the benzonitrile used revealed low levels of background benzamide even after purification. This corresponded to less than one percent of the overall quantity of benzamide formed, however this background quantity was subtracted during turnover calculations.

Table S2. Gradient GC elution conditions for benzamide.

Time (min)	Temp/Rate Change	Time	Final Temp
0	80 °C	0 min	80 °C
0–12	11.7 °C/min	12 min	220 °C
12–23	No change	11 min	220 °C

2. ^{1}H- and ^{13}C-NMR Spectra for Compounds 1–4

Figure S5. ^{1}H-NMR (300 MHz, $CDCl_3$) of Pyridine-2,6-dicarboxylic acid bis(L-cysteine methyl ester)carboxamide **1**.

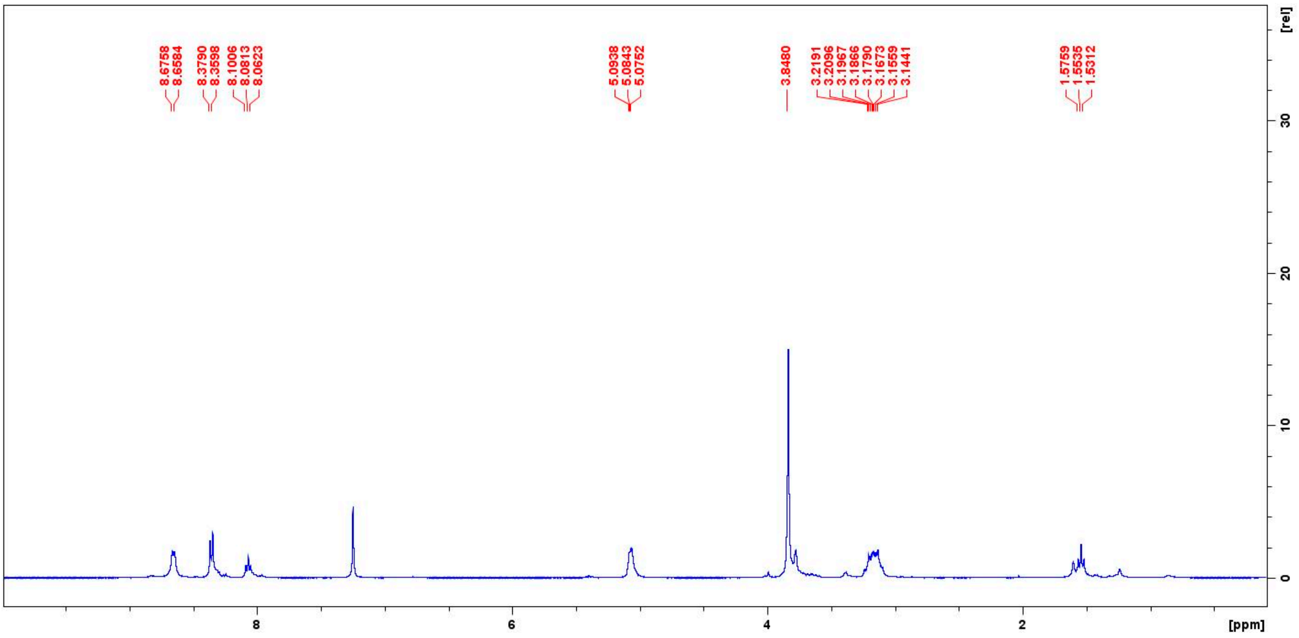

Figure S6. ^{13}C-NMR (75 MHz, $CDCl_3$) of Pyridine-2,6-dicarboxylic acid bis(L-cysteine methyl ester)carboxamide **1**.

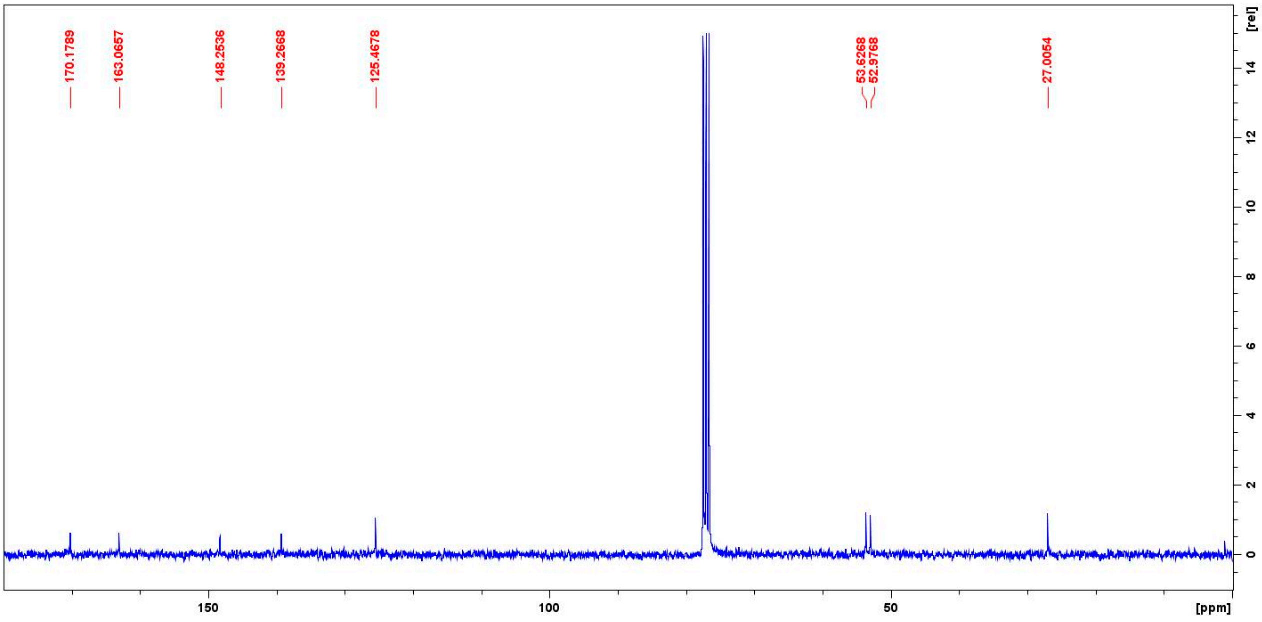

Figure S7. ^{1}H-NMR (300 MHz, $CDCl_3$) of Pyridine-2,6-dicarboxylic acid bis(*S*-methyl-L-cysteine methyl ester)carboxamide **2**.

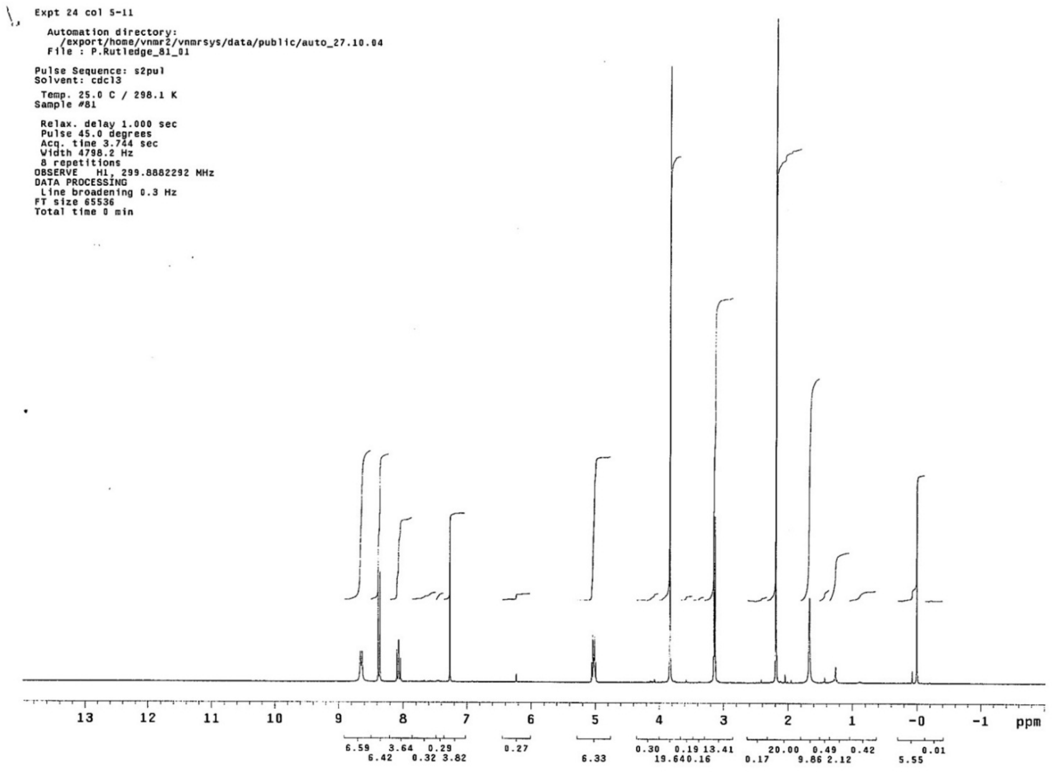

Figure S8. ^{13}C-NMR (75 MHz, $CDCl_3$) of Pyridine-2,6-dicarboxylic acid bis(*S*-methyl-L-cysteine methyl ester)carboxamide **2**.

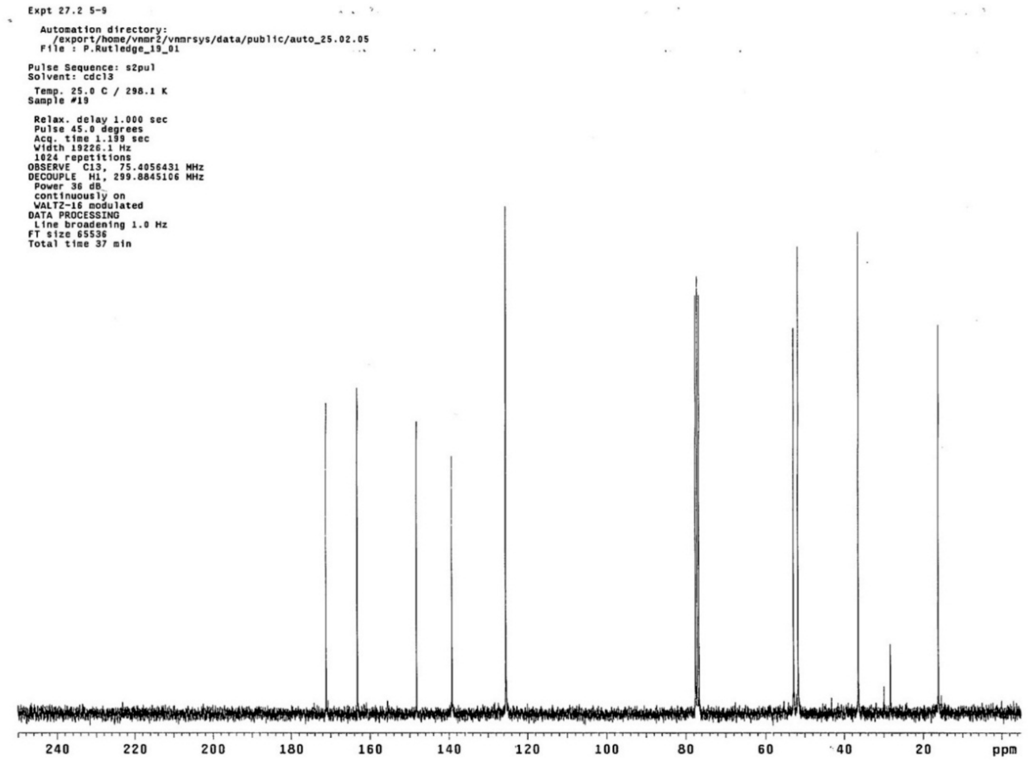

Figure S9. ^{1}H-NMR (300 MHz, $CDCl_3$) of Pyridine-2,6-dicarboxylic acid bis(L-methionine methyl ester)carboxamide **3**.

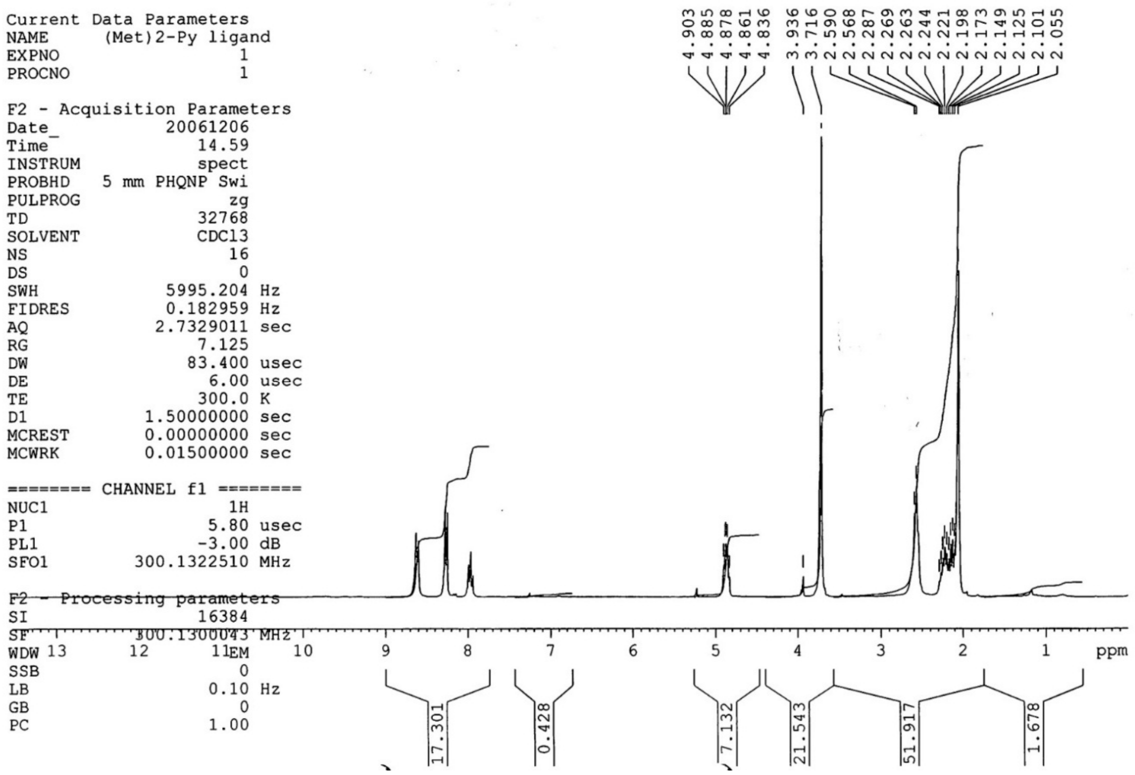

Figure S10. ^{13}C-NMR (75 MHz, $CDCl_3$) of Pyridine-2,6-dicarboxylic acid bis(L-methionine methyl ester)carboxamide **3**.

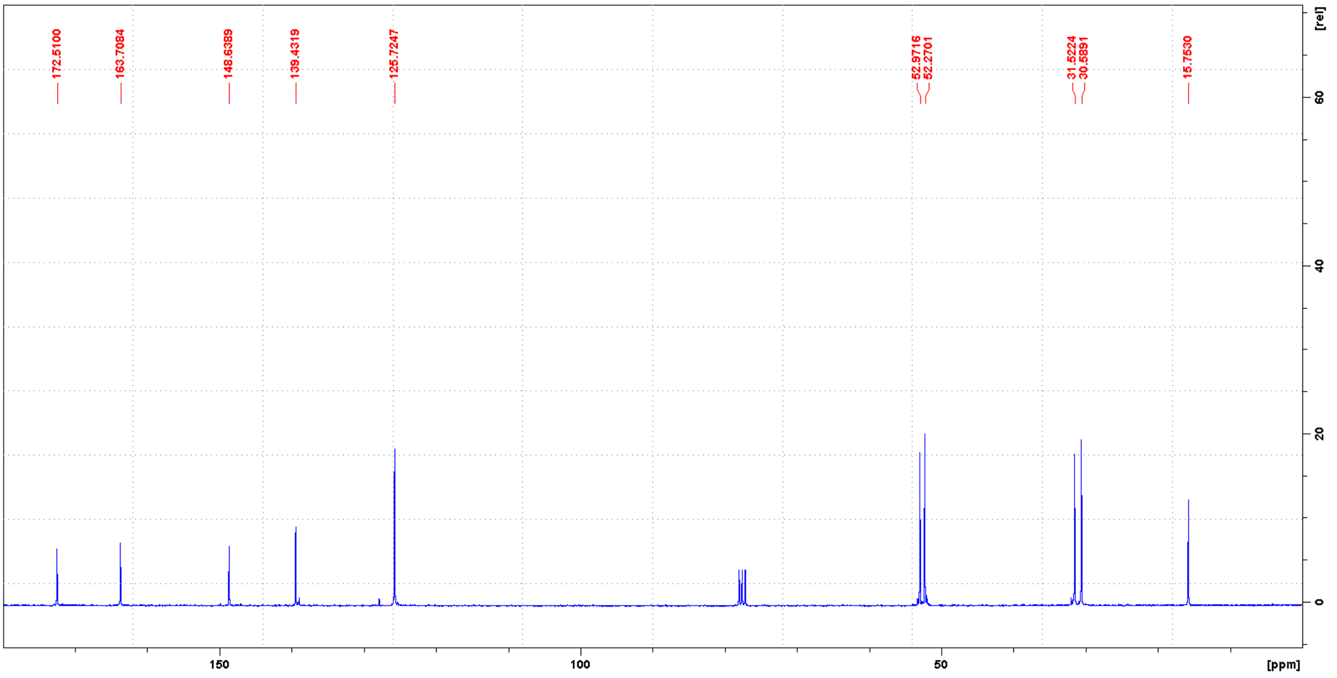

Figure S11. ^{1}H-NMR (300MHz, $CDCl_3$) of Pyridine-2,6-dicarboxylic acid bis(L-penicillamine methyl ester)carboxamide **4**.

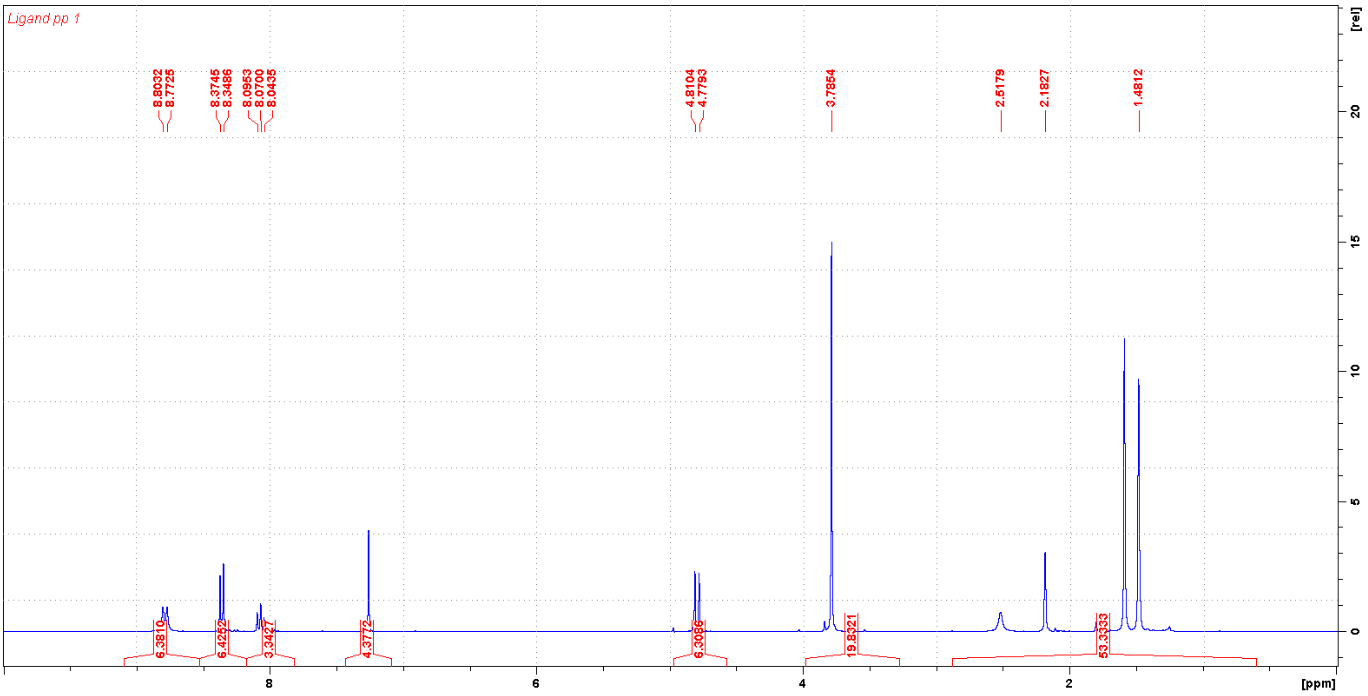

Figure S12. ^{13}C-NMR (75MHz, $CDCl_3$) of Pyridine-2,6-dicarboxylic acid bis(L-penicillamine methyl ester)carboxamide **4**.

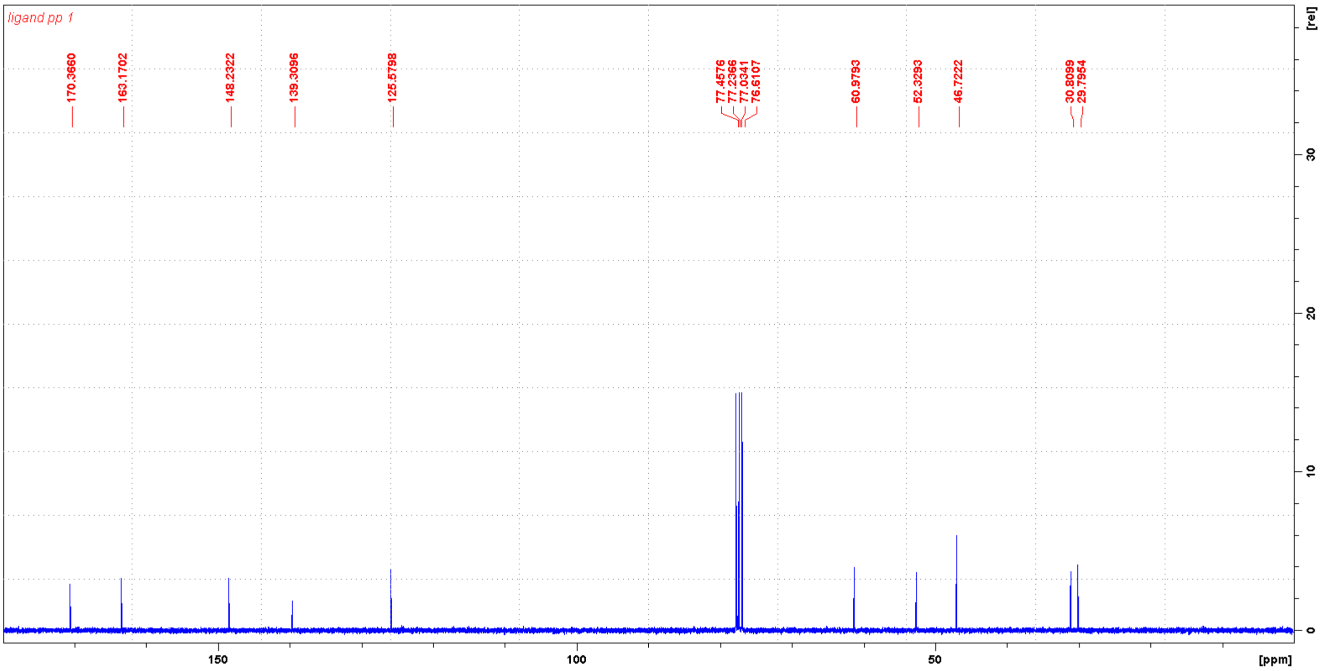

References

1. Loconto, P.R. *Trace Environmental Quantitative Analysis: Principles, Techniques and Applications*, 2nd ed.; Marcel Dekker Inc.: New York, NY, USA, 2005.

Harris, D.C. *Quantitative Chemical Analysis*, 6th ed.; W.H. Freeman: New York, NY, USA, 2003.
